# Developments in procedural sedation for adults

**DOI:** 10.1016/j.bjae.2022.02.006

**Published:** 2022-04-20

**Authors:** J.R. Sneyd

**Affiliations:** University of Plymouth, Plymouth, UK

**Keywords:** capnography, midazolam, propofol, remimazolam, sedation

**Learning objectives**

By reading this article, you should be able to:

• Describe developments in anaesthetic pharmacology relevant to procedural sedation.
• Discuss factors determining a patient's ability to return to home, work and drive.
• Recommend an appropriate starvation regimen for individual patients and their circumstances.
• Explain the use of capnography for patients receiving sedation.

**Key points**

• Capnography is now a core monitoring technique for sedation.
• Propofol, given for sedation by appropriately trained non-anaesthetists, can be safe and effective.
• Short-acting agents (remifentanil, remimazolam) and novel analgesics (oliceridine) may expand the boundaries of current practice.
• Dexmedetomidine lacks clear advantages over other agents.
• Hypotension is common during propofol sedation.

## Understanding the context of procedural sedation

Sedation is ubiquitous in clinical practice. A 2-day survey of six UK hospitals found that outside of operating theatres and ICUs, the most frequent locations where sedation was used were endoscopy, radiology and cardiology facilities and emergency departments (EDs).^1^ However, sedation is also delivered infrequently in many other environments. These minor locations may be a significant distance from the usual sources of help (emergency team, anaesthetists, intensive care), particularly in large hospitals sites. Off-site sedation is common in dental practice, typically in facilities very different from the hospital environment.

## Defining procedural sedation

Procedural sedation supports the delivery of investigations and procedures that patients might be otherwise unable to tolerate. Whereas general anaesthesia is characterised by a lack of response to surgical stimulus, minor surgical procedures supported by sedation still require effective locoregional anaesthesia. Recently, an international consensus statement defined the purpose of procedural sedation as ‘ *… to facilitate a diagnostic or therapeutic procedure*’ with a target state in which ‘ *… airway patency, spontaneous respiration, protective airway reflexes, and hemodynamic stability are preserved, while alleviating anxiety and pain*’.^2^ These carefully crafted phrases reflect a clear separation from anaesthesia and avoid ‘territorial’ claims for particular professional groups.

On an etymological note, using ‘procedural’ as an adjective in the form ‘procedural sedation’ is at odds with dictionary definitions of the word ‘procedural’ which imply something of or relating to *procedure* (rather than *a procedure*). Nevertheless, the term ‘procedural sedation’ has been increasingly adopted in the medical literature to mean sedation of patients to facilitate a diagnostic or medical procedure, and so is used in this article.

Terminology and language also affect patients' understanding. A clinician remarking ‘You'll be asleep’ or ‘You won't remember’ may set up expectations equivalent to general anaesthesia and by implication, anything less is equated to failure. Educational materials have been developed to assist discussions with patients before sedation is used.^3^

## Trends in patients, procedures and services

Excepting fee-for-service systems (where insurers may capture charges for drugs or for an anaesthetist), procedural sedation may not be recorded separately from the underpinning procedure. Data capture also varies by circumstance. Thus, modern endoscopy suites operate bespoke software, which includes some details of sedation. In contrast, a ‘one-off’ episode of sedation in the ED may be documented in the patient's notes but never become a hospital statistic. Nevertheless, the use of sedation appears to be increasing. An index procedure, colonoscopy, is typically performed with sedation. The number of colonoscopies performed in the UK increased by 13% in 2 yrs (2017–9).^4^ Colonoscopies in the EU increased by 13% in the years 2011–5 (www.ec.europa.eu/Eurostat). Setbacks from the impact of the COVID-19 pandemic on medical investigations have increased pressure to conduct procedures and investigations swiftly and efficiently on an outpatient basis.^5^

Developments in instruments, procedures and imaging, and a shift to outpatient provision have increased the volume and duration of sedation whilst offering it to a broader range of patients who may be older and have comorbidities.

## Guidelines

Guidelines for procedural sedation have been developed by specialist and professional bodies. In the UK, baseline standards were established by the Academy of Medical Royal Colleges (AoMRC) in 2013 with a minor update in 2021.^6^^,^^7^ These are accepted by all clinical groups except dentists who have acted independently.^8^ Specialty groups are encouraged to develop supplementary guidance that builds on the AoMRC's standards and interprets it into particular clinical contexts. Materials for specialty training curricula are provided. Similar initiatives have taken place in the USA and in Europe.^9^^,^^10^ British anaesthetists should ensure their familiarity with the documents produced by the AoMRC and the Association of Anaesthetists.^6^^,^^7^^,^^11^

The principal developments in sedation guidelines over the past decade reflect an emphasis on competencies rather than job descriptions qualifying an individual to give sedation. Thus, European, UK and US guidelines remind us that practitioners must achieve and sustain relevant competencies for the target state and for unplanned deeper states.^6^^,^^7^^,^^9^, ^10^, ^11^ This innocuous rescue provision is interpreted by some as grounds to prohibit the use of propofol by non-anaesthetists. Guidance from the USA also distinguishes between ‘sedative or analgesic medications not intended for general anesthesia’ and ‘sedative/analgesic medications intended for general anesthesia’.^9^

## New drugs and updates on old ones

### Remimazolam

Remimazolam is a water-soluble benzodiazepine with a rapid onset of action. The sedated state produced by remimazolam appears broadly similar to that caused by midazolam with faster onset. Because it is hydrolysed by tissue esterases, patients recover rapidly from sedation with remimazolam with psychometric measures returning to baseline sooner than with midazolam.^12^ Unlike midazolam, remimazolam metabolites have negligible sedative effects. Presented as a powder, remimazolam must be reconstituted before use. Licensed for procedural sedation in the UK, Europe and the USA, remimazolam is also licensed for induction and maintenance of anaesthesia in China, Japan and Korea.

### Oliceridine

Oliceridine is a new opioid analgesic that acts as an agonist at the μ-opioid receptor. When used intravenously for acute pain after surgery, oliceridine has been shown to be non-inferior to morphine.^13^ Whether it is a partial agonist or a biased agonist is a matter of debate, and arguably unimportant. Oliceridine may have fewer adverse effects compared with morphine – that is it has a reduced tendency to cause sedation and respiratory depression.^13^ However, the extent and clinical relevance of these differences are controversial. The drug has had a troubled path to regulatory approval and was initially refused a licence by the Food and Drug Administration (FDA) in the USA. Combinations of oliceridine–remimazolam and oliceridine–propofol have been proposed for sedation during gastrointestinal (GI) endoscopy although no trial data have been reported.^14^

### Methoxyflurane

Methoxyflurane is an old inhaled anaesthetic agent that has been repurposed as an inhaled analgesic. Now licensed in Europe for analgesia, it has also found use for sedation of patients having colonoscopy or dental extractions.^15^^,^^16^ Limiting the single dose of methoxyflurane to 3 ml (which supports up to 25–30 min of continuous use) appears to be an effective safeguard against renal injury. The manufacturers recommend no more than 6 ml in a day, none the next day and maximally 15 ml in a week.

### Sevoflurane

Sevoflurane has been evaluated for sedation during burns dressings and during painful procedures in patients without burns injuries.^17^ When 0.8% sevoflurane was inhaled for analgesia for labour pain it was more effective than nitrous oxide.^18^ However, metabolism of sevoflurane produces fluoride and hexafluoroisopropanolol (HFIP), which may accumulate in at least a proportion of patients.

Use of sevoflurane for either procedural sedation or for analgesia is off-label as the drug is only licensed for the induction and maintenance of general anaesthesia. The availability of CE marked apparatus to deliver sevoflurane in these applications does not imply either safety or efficacy. Nevertheless, the use of low-dose sevoflurane to provide brief periods of analgesia or sedation is probably safe. In contrast, the use of sevoflurane in ICU for sedation involves large doses of drug given over extended periods. This type of use appears to be associated with a demonstrable incidence of renal injury, so sedation with sevoflurane in ICU cannot be recommended.^19^

### Remifentanil

Remifentanil was evaluated for sedation during its clinical development programme.^20^ Patients having hip or hand surgery under regional block received remifentanil at 0.1, 0.07 or 0.04 μg kg^−1^ min^−1^ in a double-blind, placebo-controlled study. The median effective dose (ED_50_) to achieve an observer's assessment of alertness/sedation scale (OAA/S) derived measure of moderate sedation was estimated as 0.043 μg kg^−1^ min^−1^. Haemodynamic changes associated with remifentanil were modest. Nausea, vomiting, respiratory depression and pruritus were frequent and dose-related. Licensing for procedural sedation was not pursued, and remifentanil is only approved for sedation in patients undergoing mechanical ventilation.

A special instance of procedural sedation with remifentanil is to facilitate awake tracheal intubation. In this circumstance, the powerful antitussive effect of remifentanil enhances the patient's tolerance of airway manipulation. Furthermore, there is typically a sustained dialogue between the practitioner and the patient allowing early identification of apnoea and if necessary, encouragement of the patient to breathe.

### Dexmedetomidine

This α_2-_adrenoreceptor agonist is restricted to ‘ … health care professionals skilled in the anaesthetic management of patients in the operating room or during diagnostic procedures’ (www.medicines.org.uk). Onset of sedation is slow, and bradycardia and hypotension are frequent, especially in older people. Dexmedetomidine appears to diminish perioperative inflammatory responses in surgical patients although this may not be relevant to procedural sedation. Dexmedetomidine has been evaluated in colonoscopy. When used as the sole agent, bradycardia and hypotension were problematic and recovery was slow. When dexmedetomidine or midazolam were combined with fentanyl 1 μg kg^−1^, performance was broadly equivalent. However, the dexmedetomidine regimen was markedly slower in achieving the desired degree of sedation as it required pretreatment with a 10-min loading infusion. When compared with propofol for gastrointestinal endoscopy, dexmedetomidine was less acceptable to patients. Adding low-dose dexmedetomidine to bispectral index (BIS)-guided propofol sedation decreases propofol requirements but reduces heart rate and blood pressure whilst delaying recovery and discharge.

### Ketamine

Minimal cardiorespiratory depression and a profound analgesic effect make ketamine a candidate for procedural sedation; however, unpleasant psychiatric adverse effects prevent its use as monotherapy. Propofol–ketamine combinations have been explored and appear to have fewer adverse respiratory events when compared with propofol.^21^

### Nitrous oxide

Although standard anaesthesia machines with the ability to deliver up to 70% N_2_O are widely available, Entonox (a 50:50 mixture of O_2_ with N_2_O) is preferred for procedural sedation with nitrous oxide. Well established as a labour analgesic, Entonox is probably underused in other applications. Outwith dentistry, where it is typically used in combination with local anaesthesia, Entonox is commonly used as a sole agent. When compared with midazolam–fentanyl for sedation during colonoscopy, Entonox gave lower pain scores, faster recovery times and was preferred by patients without compromising the quality of the procedure.^22^ A similar study reported Entonox (BOC Healthcare, Manchester, UK) and propofol–fentanyl to be equally effective.^23^ Nitrous oxide is recognised as a greenhouse gas and environmental concerns have affected its use.

### Propofol

Propofol remains the drug of choice for anaesthetist-delivered procedural sedation although concerns have been raised about its tendency to cause hypotension, especially in older people.^24^ Concurrent lidocaine i.v. during propofol sedation for endoscopy reduced the propofol dose, shortened recovery time and improved endoscopists' satisfaction.^25^

Delivery options for propofol now include target-controlled infusion (TCI) and patient-controlled sedation (PCS). The use of propofol by non-anaesthetists remains controversial although it is demonstrably safe and outcomes are similar.

In the USA, where the use of propofol is limited to anaesthetists, propofol sedation underpins increased participation by anaesthesia services in outpatient colonoscopy (from 16.7% to 58.1% in the USA during the decade 2006–15) whilst having minimal impact on procedural quality indicators at considerable expense.

## Equipment for sedation

### Infusion pumps

Except in the USA (where it is not approved), TCI is the preferred means of giving propofol, and increasingly for remifentanil. Patient-controlled sedation, with repeated propofol boluses or through titration of a TCI system is associated with fewer adverse events than clinician-administered propofol whilst using similar median doses.^26^ Patient-controlled sedation is feasible and appears to be safe but the onset of sedation may be slow with implications for throughput of patients. Current commercially available infusion systems are not licensed for PCS and their use should therefore be considered experimental. Recently, a simulation study showed that TCI models for remifentanil deliver doses that lie within the range of the product licence.^27^ Given the lack of commercial incentive to develop drug licenses and equipment for generic drugs, this imaginative approach might be more broadly applicable.

### Methoxyflurane inhaler

Penthrox is a single-use device through which patients may inhale up to 3 ml methoxyflurane. Primarily intended for analgesia, the system produces a degree of sedation in a proportion of patients.^28^

## Monitoring

In addition to regularly assessing and recording the patient's sedation status, current guidelines require monitoring of arterial blood pressure, ECG and oxygen saturation.^6^^,^^7^^,^^9^, ^10^, ^11^ Recently, capnography has been upgraded to mandatory, albeit with some caveats.

### Capnography

In sedated patients the principal use of capnography is to confirm (on a continual basis) the presence of spontaneous respiration without apnoea, bradypnoea or airway obstruction. When sedation is used, exhaled breath is collected around the patient's lips and nose and transported through a fine tube to an analyser, which may be several metres away. The exhaled breath of the patient may be diluted by entrained air, which distorts the capnogram waveform and presents an apparent end-tidal CO_2_ that is lower than the true value. However, provided that a clear (albeit distorted) respiratory waveform is visible, derived values of ventilatory frequency are likely to be accurate.

**Fig. 1:**
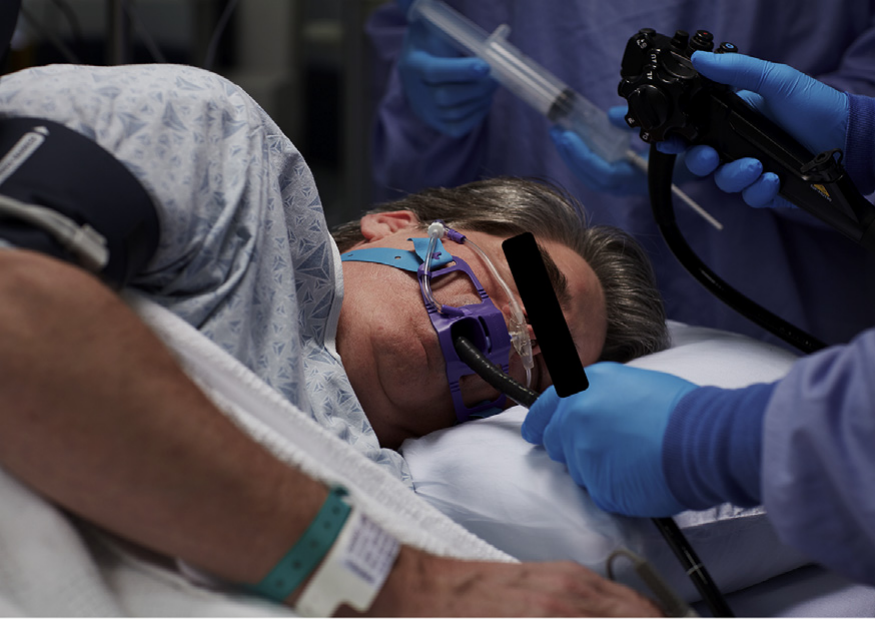
Combined bite block airway, oxygen delivery and capnography for patients undergoing upper gastrointestinal endoscopy. Separate small-bore tubes deliver oxygen and sample airway gases.

Although a useful capnogram may be achieved by tucking a sampling tube inside a standard facemask, the resulting waveform may be positional and therefore confusing. Recently, the development of soft plastics allows the manufacture of patient-friendly equipment with delivery of supplementary oxygen to the front of the nose whilst sampling within the nares and in front of the mouth. When appropriate, the incorporation of a bite guard facilitates upper GI endoscopy (Fig. 1).

Like the pulse oximeter, the capnogram waveform is intuitive and easy to understand. In contrast, interpretation of the ECG and the EEG require significant specialist training. Because the application of capnography is simple, this monitoring can easily be incorporated into routine clinical practice. Importantly, capnography has no capacity for causing harm to patients. Capnograpy during procedural sedation has been evaluated in RCTs; however, their design and analysis is heterogenous. Meta-analysis suggests that capnography reduces the number of airway incidents and interventions, with a decreased incidence of hypoxaemia.^29^ The evidence base is far from comprehensive, especially in procedures around the airway. Acceptance of capnography as a core monitor for sedated patients is general amongst anaesthetists, with slower uptake by other professional groups, notably dentists.^8^

Neither parachutes nor pulse oximeters were evaluated by clinical trials before their introduction; however, their use is widespread. Furthermore, consideration of utility only in terms of critical incidents and major outcomes may miss the point. Situational awareness errors contribute to or underpin as many as 75% of catastrophic outcomes and anaesthesia malpractice claims. A common world view is an integral element of crisis resource management and a clear understanding of the patient's status forms a priority component of this information. The combination of capnography with pulse oximetry provides the entire team with a continuous reassurance that the patient is breathing, is oxygenated and has a circulation. The availability of compact reliable monitors and well-designed apparatus to ensure effective gas sampling make capnography a logical addition to the practice of procedural sedation. Although the evidence base for capnography during sedation is moderate rather than overwhelming, its value is enhanced by supporting a safety culture in busy clinical teams.

### Bispectral index

Although it is not specified by European, UK or US guidelines, BIS monitoring may be used to confirm the degree of sedation achieved and to avoid unnecessarily deep sedation.^6^^,^^7^^,^^9^, ^10^, ^11^ Postoperative delirium is common and associated with adverse outcomes. Development of anaesthetic strategies to minimise delirium and other forms of perioperative neurocognitive disorder is an emerging research priority. Recently, BIS-guided titration of anaesthesia to target values of 50 rather than 35 for high-risk patients undergoing elective major surgery was shown to decrease the incidence of new delirium from 28% to 19%.^30^ Median average BIS scores were 51 and 38 in the two groups, respectively. In contrast, when patients having lumbar spine fusions were randomised to receive either BIS sedation or unguided general anaesthesia with median average BIS values of 62 and 45, respectively, the incidence of delirium was not different.^31^ However, a preplanned sub-group analysis did demonstrate benefit in patients with preoperative cognitive impairment. As many surgeries can be performed with locoregional anaesthesia and procedural sedation rather than general anaesthesia, there is continued effort to clarify whether these techniques can affect the incidence and severity of perioperative neurocognitive disorders. Although clinicians may find BIS monitoring useful, its use remains controversial. Certainly, it remains unproven whether BIS monitoring can decrease the incidence of perioperative neurocognitive disorders.

## Haemodynamic monitoring

Improvements in engineering and signal processing allow non-invasive extraction and display of arterial pressure waveforms. Further analysis yields a predictive index that forecasts hypotension occurring. Currently deployed for major cases, we may expect to see this and similar technologies deployed progressively in standard monitors.

## Hypotension

The recognition that perioperative hypotension is associated with adverse outcomes has made its identification and avoidance a clinical priority. In contrast, changes in blood pressure during procedural sedation have received little attention. Propofol sedation is particularly problematic, and hypotension is frequent when propofol is used for sedation of patients having colonoscopy.^24^ Furthermore, its hypotensive effects are amplified by haemorrhage. Barends and colleagues^32^ reported 2973 episodes of sedation provided by specialist nurses using TCIs of propofol and remifentanil. ‘Significant’ hypotension (mean arterial pressure <65 mmHg for longer than 10 min and requiring treatment) occurred in 8.8% of patients. For the 286 undergoing lower endoscopy, the incidence was 12.9%. The occurrence of material hypotension during procedural sedation is thus established. Further work is required to establish its determinants and significance. Gregory and colleagues^33^ make the case for intraoperative hypotension as a crucial target ‘ … we believe hypotension in the operating room is a serious public health issue, and should not be ignored in any age group’. ‘We suggest there is an urgent and currently unmet need for prospective interventional studies focused on its prevention’.^33^ Arguably, it is time that hypotension during procedural sedation received the same attention as its equivalent in the operating room.

## Fasting

**Fig. 2:**
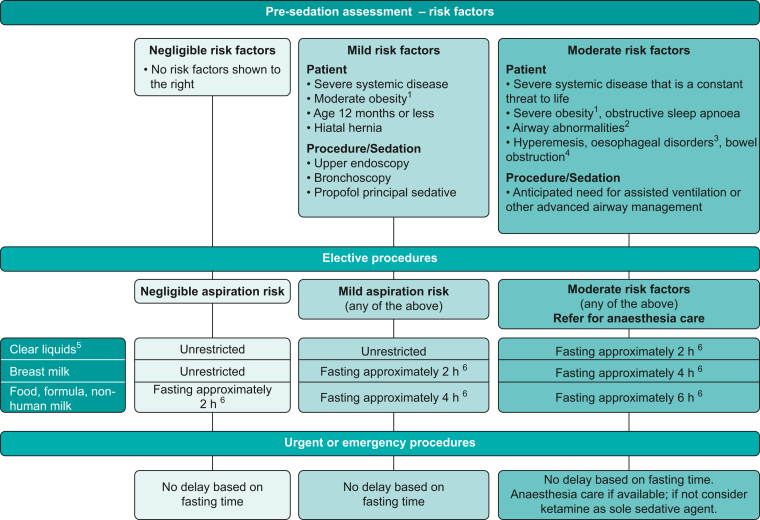
Algorithm linking risk stratification and fasting guidance. Notes: (1) Suggested definitions for moderate obesity are a BMI of 30–39 kg m^−2^ in adults or from the 85th up to the 95th BMI percentile based on age/sex in a child, and for severe obesity a BMI of 40 kg m^−2^ or higher in an adult or at the 95th percentile or greater in a child. (2) Includes micrognathia, macroglossia and laryngomalacia. (3) Includes gastroparesis, achalasia, atresia, stricture and tracheo-oesophageal fistula. (4) Includes ileus, pseudo-obstruction, pyloric stenosis and intussusception. (5) Clear liquids are generally considered to include water, fruit juices without pulp, clear tea, black coffee and specially prepared carbohydrate-containing fluids. (6) Fasting intervals are not absolute, with exceptions permissible when the volumes of oral intake are minor, or the fasting time reasonably close. Reproduced with permission from Green and colleagues,^34^ © 2019 John Wiley and Sons, Inc.

A recent international consensus statement on fasting before procedural sedation concluded that ‘the probability of clinically important aspiration during procedural sedation is negligible’. And ‘current concerns about aspiration are out of proportion to the actual risk’.^34^ An algorithm-based approach to decision-making is recommended (Fig. 2). The algorithm attempts to pragmatically recognise that needless fasting inconveniences patients and staff whilst acknowledging the fine line between sedation and anaesthesia. In addition, a small minority of procedures where sedation is planned may evolve into needing anaesthesia.

## Recovery from sedation and driving

Patients undergoing sedation require clear guidance on the timing of their discharge from a clinical facility, the need for an escort, and the appropriate intervals before driving, operating machinery, or making important decisions. Although surgical considerations or the persistence of regional blocks may determine some of these recommendations the principal consideration is residual psychometric effect from hypnotic drugs and opioids. In the UK, colonoscopy patients self-sedated with 50% nitrous oxide (Entonox) are considered safe to drive home 30 min later. The application of ‘soft’ pharmacology to procedural sedation through the tissue esterase-hydrolysed agents remifentanil and remimazolam offers the prospect of swift recovery to baseline cognitive capability but is not yet validated to a degree sufficient to justify changed clinical practice.

Current guidance derives from pharmaceutical companies (drug data sheets), national bodies, specialty associations and local practice. Although research using driving simulators and psychometric testing can suggest where there may be scope for relaxation of guidance, there is little evidence of its implementation. The difficulty of proving a negative – that is that an individual is unimpaired – and an abundance of caution represent substantial obstacles to progress in this area.^35^

## Audit and adverse event reporting

An initiative to support the capture of coherent and comparable data describing episodes of procedural sedation has developed tools for quality improvement and research.^36^ These have been deployed by ED physicians to demonstrate their safe practice in the sedation of adults, children and older people.

## Current and future practice and staffing

The predominant current sedation strategies are hypnotic–opioid combinations. Anaesthetists typically provide deep sedation or general anaesthesia using propofol as a hypnotic supplemented by an opioid, usually remifentanil or fentanyl. Non-anaesthetist operators giving sedation most frequently use midazolam–fentanyl.^1^

In the USA, provision by anaesthetists of propofol sedation for colonoscopy has grown rapidly but its cost and necessity are being challenged. ‘Non-anaesthetist administration of propofol’ (NAAP) is restricted by drug labels reserving propofol for certain professional groups (in the USA) and the reluctance of non-anaesthetists to use an anaesthetic agent or by law (in some European countries). In addition, traditional interprofessional boundaries and custom and practice have restrained the uptake of NAAP. However, overall efficiency may be greater with NAAP. Non-anaesthetist (nurse) use of propofol may even be preferable to use by anaesthetists; when nurses use propofol for sedation, they use less drug and cause less hypotension than do anaesthetists in the same institution.^37^

## The future

Looking ahead, the demand for sedation will probably continue to exceed the capacity of anaesthetists to provide it. Where non-anaesthetist colleagues are providing sedation, anaesthetists are well positioned to support them through standards, training, simulation and audit. Anaesthetists should be at the centre of hospital sedation practice with key roles at institutional levels including Sedation Committees, Formulary Committees and general support for pharmacovigilance and patients' safety. Hospital-wide sedation services provided by anaesthesia departments and using non-medical practitioners have been established in the Netherlands and elsewhere and appear to be safe.

## Conclusions

New drugs and equipment, and developments in policy and procedure offer opportunities to improve our practice in procedural sedation.

## Declaration of interests

JRS acts as consultant and Advisory Board member for Paion and as consultant for Medtronic.

## MCQs

The associated MCQs (to support CME/CPD activity) will be accessible at www.bjaed.org/cme/home by subscribers to *BJA Education*.
