# Supplementary material for: Developments in procedural sedation for adults
Source: BJA Educ. 2022 Apr 20;22(7):258–64. doi: 10.1016/j.bjae.2022.02.006 (PMC9214437; doi:10.1016/j.bjae.2022.02.006)
Supplement: Multimedia component 1 [file mmc1.docx]

READING LIST

Context

Leslie K, Allen ML, Hessian EC, et al. Safety of sedation for gastrointestinal endoscopy in a group of university-affiliated hospitals: a prospective cohort study. *Br J Anaesth* 2017; **118**: 90-9

Sneyd JR, Montgomery H, Pencheon D. The anaesthetist and the environment. *Anaesthesia* 2010; **65**: 435-7

Yeh RW, Valsdottir LR, Yeh MW, et al. Parachute use to prevent death and major trauma when jumping from aircraft: randomized controlled trial. *BMJ* 2018: k5094

Drugs

Abu Baker F, Mari A, Aamarney K, Hakeem AR, Ovadia B, Kopelman Y. Propofol sedation in colonoscopy: from satisfied patients to improved quality indicators. *Clin Exp Gastroenterol* 2019; **12**: 105-10

Alburquerque M, Smarrelli A, Montesinos JC, et al. Outcomes of colonoscopy with non-anesthesiologist-administered propofol (NAAP): an equivalence trial. *Endoscopy international open* 2021; **09**: E1070-E6

Azzam AAH, McDonald J, Lambert DG. Hot topics in opioid pharmacology: mixed and biased opioids. *Br J Anaesth* 2019; **122**: e136-e45

Ball AJ, Campbell JA, Riley SA. Nitrous oxide use during colonoscopy: a national survey of English screening colonoscopists. *Frontline Gastroenterol* 2014; **5**: 254-9

Bergese SD, Brzezinski M, Hammer GB, et al. ATHENA: A Phase 3, Open-Label Study Of The Safety And Effectiveness Of Oliceridine (TRV130), A G-Protein Selective Agonist At The μ-Opioid Receptor, In Patients With Moderate To Severe Acute Pain Requiring Parenteral Opioid Therapy. *J Pain Res* 2019; **12**: 3113-26

Borrat X, Ubre M, Risco R, et al. Computerized tests to evaluate recovery of cognitive function after deep sedation with propofol and remifentanil for colonoscopy. *J Clin Monit Comput* 2019; **33**: 107-13

Dere K, Sucullu I, Budak ET, et al. A comparison of dexmedetomidine versus midazolam for sedation, pain and hemodynamic control, during colonoscopy under conscious sedation. *Eur J Anaesthesiol* 2010; **27**: 648-52

Doi M, Morita K, Takeda J, Sakamoto A, Yamakage M, Suzuki T. Efficacy and safety of remimazolam versus propofol for general anesthesia: a multicenter, single-blind, randomized, parallel-group, phase IIb/III trial. *J Anesth* 2020

Edokpolo LU, Mastriano DJ, Serafin J, Weedon JC, Siddiqui MT, Dimaculangan DP. Discharge Readiness after Propofol with or without Dexmedetomidine for Colonoscopy: A Randomized Controlled Trial. *Anesthesiology* 2019; **131**: 279-86

Facciorusso A, Turco A, Barnabà C, Longo G, Dipasquale G, Muscatiello N. Efficacy and Safety of Non-Anesthesiologist Administration of Propofol Sedation in Endoscopic Ultrasound: A Propensity Score Analysis. *Diagnostics* 2020; **10**: 791

Hewson DW. Counting the costs of patient‐led propofol sedation. *Acta Anaesthesiol Scand* 2021; **65**: 278-

Ikeda S. The Reincarnation of Methoxyflurane. *J Anesth Hist* 2020; **6**: 79-83

Johnson KB, Egan TD, Kern SE, et al. The Influence of Hemorrhagic Shock on Propofol: A Pharmacokinetic and Pharmacodynamic Analysis. *Anesthesiology* 2003; **99**: 409-20

Karanth H, Murali S, Koteshwar R, Shetty V, Adappa K. Comparative Study between Propofol and Dexmedetomidine for Conscious Sedation in Patients Undergoing Outpatient Colonoscopy. *Anesth Essays Res* 2018; **12**: 98-102

Kidd LR, Lyons SC, Lloyd G. Paediatric procedural sedation using ketamine in a UK emergency department: a 7 year review of practice. *Br J Anaesth* 2016; **116**: 518-23

L'Heudé M, Poignant S, Elaroussi D, Espitalier F, Ferrandière M, Laffon M. Nephrogenic diabetes insipidus associated with prolonged sedation with sevoflurane in the intensive care unit. *British Journal of Anaesthesia* 2019; **122**: e73-e5

Lambert D, Calo G. Approval of oliceridine (TRV130) for intravenous use in moderate to severe pain in adults. *Br J Anaesth* 2020; **125**: e473-e4

Maslekar S, Gardiner A, Hughes M, Culbert B, Duthie GS. Randomized clinical trial of Entonox® versus midazolam–fentanyl sedation for colonoscopy. *Br J Surg* 2009; **96**: 361-8

Newstead B, Bradburn S, Appelboam A, et al. Propofol for adult procedural sedation in a UK emergency department: safety profile in 1008 cases. *Br J Anaesth* 2013; **111**: 651-5

Nishizawa T, Suzuki H, Hosoe N, Ogata H, Kanai T, Yahagi N. Dexmedetomidine vs propofol for gastrointestinal endoscopy: A meta-analysis. *United European Gastroenterology Journal* 2017; **5**: 1037-45

Pastis NJ, Yarmus LB, Schippers F, et al. Safety and Efficacy of Remimazolam Compared With Placebo and Midazolam for Moderate Sedation During Bronchoscopy. *Chest* 2019; **155**: 137-46

Perbet S, Bourdeaux D, Lenoire A, et al. Sevoflurane for procedural sedation in critically ill patients: A pharmacokinetic comparative study between burn and non-burn patients. *Anaesthesia Critical Care & Pain Medicine* 2018; **37**: 551-6

Rahman SM, Quinn E. BET 1: Green or blue for you? Methoxyflurane (Penthrox) or nitrous oxide/oxygen 50% mixture (Entonox) for the management of acute pain in the ED. *Emergency Medicine Journal* 2019; **36**: 506-8

Viscusi ER, Skobieranda F, Soergel DG, Cook E, Burt DA, Singla N. APOLLO-1: a randomized placebo and active-controlled phase III study investigating oliceridine (TRV130), a G protein-biased ligand at the &micro;-opioid receptor, for management of moderate-to-severe acute pain following bunionectomy. *J Pain Res* 2019; **12**: 927-43

Viscusi ER, Webster L, Kuss M, et al. A randomized, phase 2 study investigating TRV130, a biased ligand of the μ-opioid receptor, for the intravenous treatment of acute pain. *Pain* 2016; **157**: 264-72

Wang K, Wu M, Xu J, et al. Effects of dexmedetomidine on perioperative stress, inflammation, and immune function: systematic review and meta-analysis. *Br J Anaesth* 2019; **123**: 777-94

Wiltshire HR, Kilpatrick GJ, Tilbrook GS, Borkett KM. A placebo- and midazolam-controlled phase I single ascending-dose study evaluating the safety, pharmacokinetics, and pharmacodynamics of remimazolam (CNS 7056): Part II. Population pharmacokinetic and pharmacodynamic modeling and simulation. *Anesth Analg* 2012; **115**: 284-96

Equipment

Conway A, Douglas C, Sutherland JR. A systematic review of capnography for sedation. *Anaesthesia* 2016; **71**: 450-4

Grossmann B, Nilsson A, Sjöberg F, Nilsson L. Patient-controlled Sedation During Flexible Bronchoscopy: A Randomized Controlled Trial. *Journal of Bronchology & Interventional Pulmonology* 2020; **27**: 77-85

Hatib F, Jian Z, Buddi S, et al. Machine-learning algorithm to predict hypotension based on high-fidelity arterial pressure waveform analysis. *Anesthesiology* 2018; **129**: 663-74

Maslekar S, Balaji P, Gardiner A, Culbert B, Monson JRT, Duthie GS. Randomized controlled trial of patient-controlled sedation for colonoscopy: Entonox vs modified patient-maintained target-controlled propofol. *Colorectal Dis* 2011; **13**: 48-57

Myles PS, Leslie K, McNeil J, Forbes A, Chan MT. Bispectral index monitoring to prevent awareness during anaesthesia: the B-Aware randomised controlled trial. *Lancet* 2004; **363**: 1757-63

Parker W, Estrich CG, Abt E, et al. Benefits and harms of capnography during procedures involving moderate sedation: A rapid review and meta-analysis. *J Am Dent Assoc* 2018; **149**: 38-50 e2

Saunders R, Struys MMRF, Pollock RF, Mestek M, Lightdale JR. Patient safety during procedural sedation using capnography monitoring: a systematic review and meta-analysis. *BMJ Open* 2017; **7**: e013402

Audit & Governance

Evered L, Silbert B, Knopman DS, et al. Recommendations for the nomenclature of cognitive change associated with anaesthesia and surgery-2018. *Br J Anaesth* 2018; **121**: 1005-12

Goudra B, Singh PM, Lichtenstein GR. Medical, Political, and Economic Considerations for the Use of MAC for Endoscopic Sedation: Big Price, Little Justification? *Dig Dis Sci* 2020; **65**: 2466-72

Homfray G, Palmer A, Grimsmo-Powney H, Appelboam A, Lloyd G. Procedural sedation of elderly patients by emergency physicians: a safety analysis of 740 patients. *Br J Anaesth* 2018; **121**: 1236-41

Krigel A, Chen L, Wright JD, Lebwohl B. Substantial Increase in Anesthesia Assistance for Outpatient Colonoscopy and Associated Cost Nationwide. *Clin Gastroenterol Hepatol* 2019; **17**: 2489-96

Mason KP, Green SM, Piacevoli Q. Adverse event reporting tool to standardize the reporting and tracking of adverse events during procedural sedation: a consensus document from the World SIVA International Sedation Task Force. *Br J Anaesth* 2012; **108**: 13-20

Ooi M, Thomson A. Morbidity and mortality of endoscopist-directed nurse-administered propofol sedation (EDNAPS) in a tertiary referral center. *Endoscopy international open* 2015; **3**: E393-7

Riesco-López JM, Rizo-Pascual J, Díaz-Sánchez A, et al. Endoscopist-directed propofol is more efficient than anesthesiologist-administered propofol in patients at low-intermediate anesthetic risk. *Eur J Gastroenterol Hepatol* 2020; **32**: 1440-6

Schoonjans C, Tate DJ. Endoscopist-administered propofol sedation during colonoscopy: Time to take over the syringe? *Gastrointest Endosc* 2021; **93**: 209-11

Schulz CM, Burden A, Posner KL, et al. Frequency and Type of Situational Awareness Errors Contributing to Death and Brain Damage. *Anesthesiology* 2017; **127**: 326-37

Sieg A, bng-Study-Group, Beck S, et al. Safety analysis of endoscopist-directed propofol sedation: a prospective, national multicenter study of 24 441 patients in German outpatient practices. *J Gastroenterol Hepatol* 2014; **29**: 517-23

Sneyd JR. Making sense of propofol sedation for endoscopy. *Br J Anaesth* 2017; **118**: 6-7
